# Intracellular Adhesion Molecule-1 K469E Gene Polymorphism and Risk of Diabetic Microvascular Complications: A Meta-Analysis

**DOI:** 10.1371/journal.pone.0069940

**Published:** 2013-07-26

**Authors:** Xianghui Su, Xi Chen, Lei Liu, Xiangyun Chang, Xuefeng Yu, Kan Sun

**Affiliations:** 1 Department of Endocrinology, Tongji Hospital, Tongji Medical College, Huazhong University of Science and Technology, Wuhan, People’s Republic of China; 2 Department of Endocrinology, First Hospital Affiliated to Medical College of Shihezi University, Xinjiang, People’s Republic of China; 3 Department of Internal Medicine,Tongji Hospital, Tongji Medical College, Huazhong University of Science and Technology, Wuhan, People’s Republic of China; Medical University Innsbruck, Austria

## Abstract

**Background:**

A number of studies evaluated the association of intracellular adhesion molecule-1 (*ICAM-1*) K469E (rs5498, A/G) gene polymorphism with diabetic microvascular complications (DMI) including diabetic nephropathy (DN) and diabetic retinopathy (DR) in different populations. However, the results of individual studies remain conflicting.

**Methods:**

A comprehensive search was conducted to identify all eligible studies of the above-mentioned associations. The pooled odds ratios (ORs) and 95% confidence intervals (CIs) were assessed using the fixed or random effect model.

**Results:**

Seven studies involving 3411 subjects were included. Overall, the meta-analysis showed a significant association of the A allele with increased risk of DMI susceptibility in a recessive model (OR = 1.37, 95% CI 1.04–1.80, *P* = 0.02). In the subgroup analysis stratified by ethnicity, significant association was found in Asians but not in Caucasians (OR = 1.78, 95% CI 1.13–2.81, *P* = 0.01; OR = 1.10, 95% CI 0.79–1.54, *P* = 0.58, respectively). Moreover, it showed a significant association between the A allele and risk of DN in a recessive model (OR = 1.25, 95% CI 1.02–1.55, *P* = 0.04).

**Conclusions:**

This meta-analysis suggested that the K469E polymorphism in *ICAM-1* gene might affect individual susceptibility to DMI and showed a discrepancy in different ethnicities. Further investigations are needed to validate the association.

## Introduction

There has been a rising epidemic of diabetes mellitus throughout the world in recent years and an alarming increase in the rate of mortality and morbidity due to coexisting dyslipidemia, atherosclerosis and cardiovascular disease [1]. It has been known that diabetic patients often develop macrovascular and/or microvascular complications. Although long-term complications of diabetes develop gradually, they can eventually be disabling or even life threatening. Diabetic microvascular complications (DMI), mainly including diabetic nephropathy (DN) and diabetic retinopathy (DR), have become major causes of chronic kidney disease and blindness [2]. Moreover, diabetic microvascular complication patients have been shown to have higher mortality among diabetic subjects [3], [4]. Both DN and DR are wide ranging and are due at least in part to chronic elevation of blood glucose levels, which leads to damage of smallest blood vessels. The pathophysiology of DN and DR are more or less similar, which commence with increase in vascular permeability. Despite the exact mechanism of DMI has not so far been clearly described, many risk factors, like the duration of diabetes, degree of glycemic control and age of the patient, are identified in causation of DMI [5], [6]. Genetic susceptibility has also been suggested as one of the reasons proven in previous studies [7], [8], [9].

Intercellular adhesion molecule-1 (*ICAM-1*), a cell surface glycoprotein, is a member of the immunoglobulin superfamily of adhesion molecules [10], [11]. It mediates adhesion of circulating leukocytes to the activated endothelium, which is one of the earliest events in the pathogenesis of inﬂammation and atherosclerosis [12]. Many lines of evidence, ranging from in vitro experiments and pathological examinations to epidemiological studies, have shown that inﬂammation is a cardinal pathogenic mechanism in diabetic microvascular diseases [13]. Although the precise role of inflammation in the development of diabetic microvascular diseases is still unclear, it is likely that inflammation can accelerate atherosclerosis in patients with diabetes [14]. Several studies have documented an increase in expression of *ICAM-1* in animal models of diabetic nephropathy and diabetic retinopathy [15], [16], [17]. The single nucleotide G to A polymorphism in the sixth exon of the *ICAM-1* gene (K469E, rs5498) results in an amino acid substitution from glutamic acid (E) to lysine (K) in immunoglobulin-like domain 5 of the *ICAM-1* protein. It has been shown to inﬂuence the binding of ICAM-1 on endothelial cells and lymphocyte function-associated antigen-1 (LFA-1) and Mac-1 on leucocytes, mediating leukocytosis and its migration in an inﬂammatory environment [18], [19]. Recent genome wide association study has demonstrated a strong correlation between K469E polymorphism and sICAM-1 levels [20]. This polymorphism has been reported to be involved in inflammatory related diseases including DMI [21], [22], [23].

Although several studies investigated the association between *ICAM-1* gene K469E polymorphism and DMI, their results remain inconsistent [23], [24], [25], [26], [27], [28], [29]. Considering a single study may lack the power to provide dependable conclusion, we carried out a meta-analysis to evaluate the precise effect of K469E polymorphism in *ICAM-1* gene on risk of DMI including DN and DR. This was, to our knowledge, the first meta-analysis of the association between *ICAM-1* gene K469E polymorphism and DMI susceptibility.

## Materials and Methods

### Publication Search

The electronic databases PubMed, Embase and Web of Science (ISI) were searched using the following terms: “*ICAM-1″*, “diabetic retinopathy” or “diabetic nephropathy”, “mutation” or “single nucleotide polymorphism (SNP)” or “variant”. Both type 1 and type 2 diabetic patients were enrolled in the study. Additional studies not captured by our database searches were identified through reviewing the reference lists of retrieved articles. The included studies were published ranging from 2002 to 2012 (last research was updated on January, 2013).

### Inclusion Criteria

Two investigators reviewed all identified articles independently to determine whether an individual study was eligible for inclusion in our meta-analysis. The inclusion criteria were as follows: (1) case-control study published as an original study to evaluate the association between K469E polymorphism in *ICAM-1* gene and risk of DN and/or DR; (2) available genotype distribution of cases and controls that can provide sufficient data for calculation; (3) published in English.

### Data Extraction

All data were collected according to a standard technological process. Studies that were repeated, did not satisfy the inclusion criteria, and provided little information or insufficient data were excluded. Table 1 lists the characteristics of the extracted data, including the name of the first author, publication year, ethnicity of the study population, number in case and control groups, genotype distributions, mean age, female sex percentage in case and control groups. We verified accuracy of data by comparing collection forms from two independent investigators. Any discrepancy between the two investigators was resolved by the third investigator.

**Table 1:** Study Characteristics of genotypes in DMI cases and controls in the analysis of *ICAM-1* gene K469E polymorphism.

Author	Year	Population	Case type	Genotypes (AA/AG/GG)				Mean age(Case/Control)
				Case		Control		
				Number (%)	*P*h*	Number (%)	*P*h*	
Kamiuchi et al.	2002	Japanese	DR	34/35/12 (42/43/15)	0.55	10/30/10 (20/60/20)	0.16	64.3/64.1
Liu et al.	2006	Chinese	DR	81/40/11 (61/30/9)	0.07	16/15/9 (40/38/22)	0.15	–
Ma et al.	2006	Swedish	DN	54/117/25 (27/60/13)	<0.05	50/156/28 (21/67/12)	<0.05	46/44
Ma et al.	2008	Swedish	DN	210/311/141 (32/47/21)	0.20	171/326/121 (28/53/19)	0.12	44/40
Petrovic et al.	2008	Caucasian	DR	47/96/52 (24/49/27)	0.84	44/77/22 (31/54/15)	0.22	65/67
Balasubbu et al.	2010	Indian	DR	103/162/80 (30/47/23)	0.29	99/174/86 (28/48/24)	0.58	57/59
Vinita et al.	2012	Indian	DR	60/92/47 (30/46/24)	0.31	29/84/44 (18/54/28)	0.32	58.8/64.3

Abbreviations and definitions: DR, diabetic retinopathy; DN, diabetic nephropathy.

* Exact *p* value for HWE test.

### Statistical Analysis

The relationship between K469E (A/G) polymorphism and DMI including DR and DN was compared using odds ratios (ORs) and their corresponding 95% confidence intervals (95% CIs). The Z test was used to determine the pooled OR with the significance level set at *P*<0.05. Dominant, recessive and additive genetic models were all used in our meta-analysis. The *I^2^* of Higgins and Thompson was used to test the heterogeneity between the studies [30]. In the presence of substantial heterogeneity (*I^2^*>50%) [31], the random-effects model (REM) by the DerSimonian and Laird method was adopted as the pooling method. Otherwise, the pooled OR was estimated using the fixed-effects model (FEM).

An exact Chi-square test was used to assess the Hardy–Weinberg equilibrium (HWE) with the significance level set at *P*<0.05. The potential publication bias was evaluated by Egger’s test and visual inspection of funnel plots. All statistical analyses were performed using the STATA version 11.0 software (Stata Corporation, College Station, TX, USA).

## Results

### Studies and Populations

**Figure 1:**
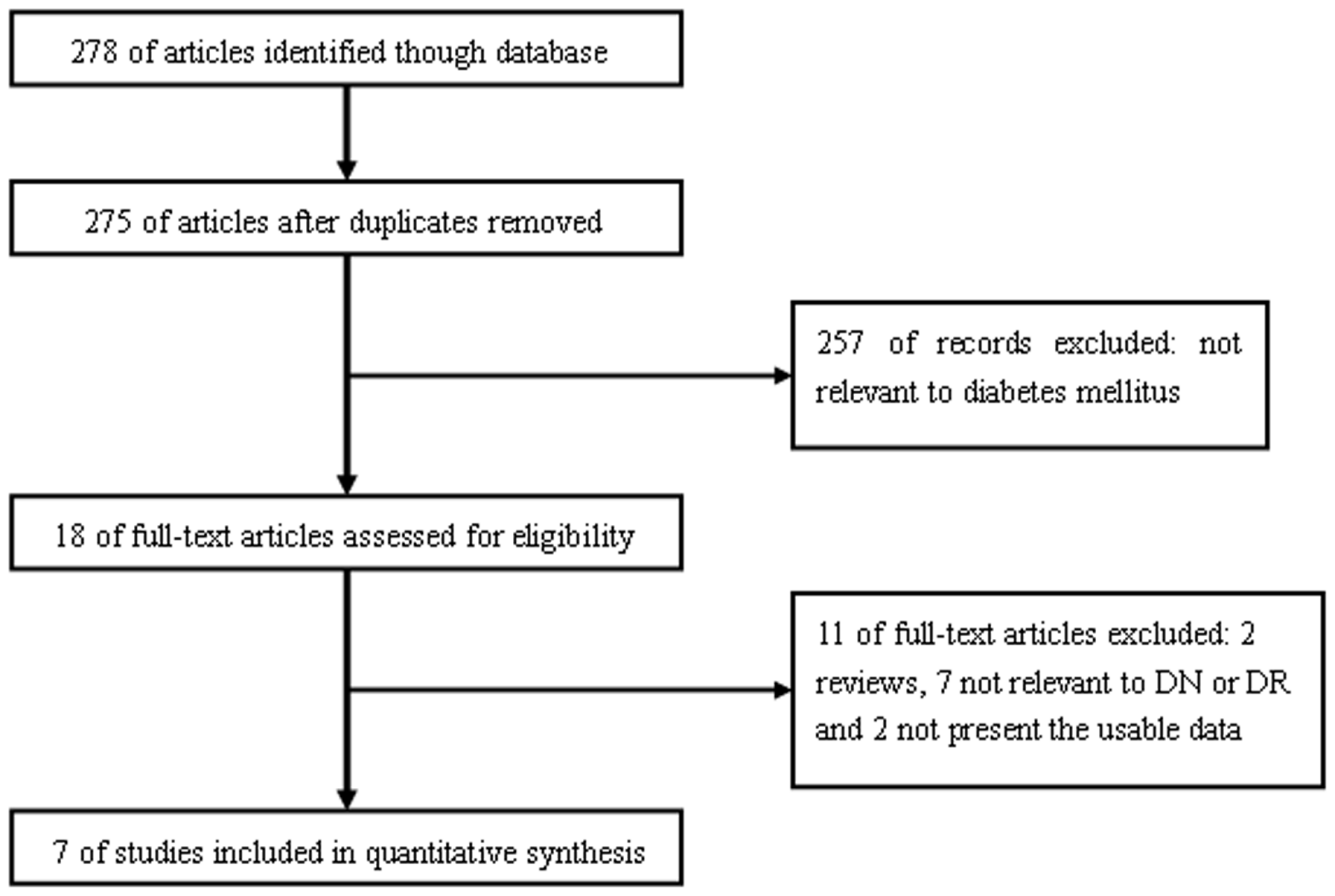
The flow chart of literature search and study selection.

Figure 1 outlines our study selection process. Briefly, a total of 278 articles were identified after an initial search. After reading the abstracts, we removed 3 duplications and 257 articles not relevant to diabetes. After reading full texts of the remaining 18 articles, 11 were then excluded. Finally, a total of 7 case-control studies in 7 articles were identified met our inclusion criteria [23], [24], [25], [26], [27], [28], [29], including 1810 DMI patients and 1601 diabetic controls. The general characteristics of each study, genotype numbers, and HWE examination results are presented in Table 1.

### Quantitative Data Synthesis

#### All studies

**Figure 2:**
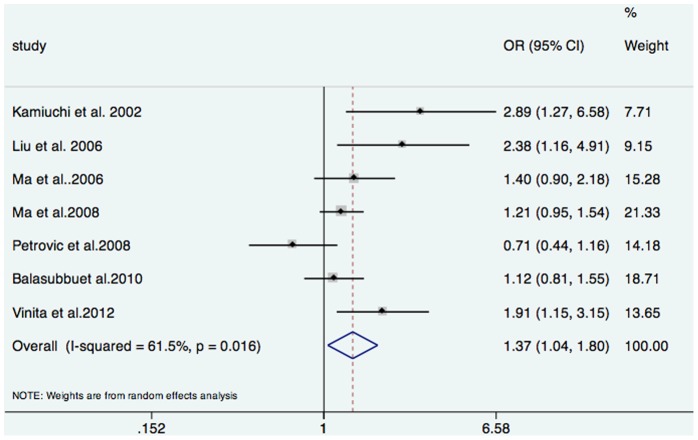
Forest plot of the overall risk of DMI associated with the *ICAM-1* gene K469E polymorphism.

As shown in Figure 2, a significant association between the *ICAM-1* K469E gene polymorphism and DMI was found in a recessive genetic model (OR = 1.37, 95% CI 1.04–1.80, *P* = 0.02). However, no significant association was found either using a dominant model (OR = 1.02, 95% CI 0.76–1.36, *P* = 0.88) or an additive model (OR = 1.17, 95% CI 0.95–1.43, *P* = 0.13) in all study populations (Table 2). In the analysis of heterogeneity, all the *I^2^* values of three models for 7 studies were higher than 50% (recessive model: 61.5%, dominant model: 56.1%, and additive model: 72.5%, respectively). Therefore, the random-effects model was used for synthesis of the data.

**Table 2:** Pooled measures on the relation of *ICAM-1* gene K469E polymorphism with DMI, DR and DN.

Group	Data	Inherited model	Number of cases/controls	Pooled OR (95% CI)	*I* ^2^ (%)
All	All relevant articles	Dominant	1770/1601	1.02 (0.77–1.36)	56.1
		Recessive	1770/1601	1.37 (1.04–1.80)*	61.5
		Additive	1770/1601	1.17 (0.95–1.43)	72.5
	Excluded for DHWE	Dominant	1574/1367	1.05 (0.75–1.46)	63.4
		Recessive	1574/1367	1.38 (1.00–1.91)*	67.5
		Additive	1574/1367	1.19 (0.93–1.52)	77.0
Asian	All relevant articles	Dominant	757/606	1.22 (0.94–1.58)	36.6
		Recessive	757/606	1.79 (1.13–2.81)*	62.5
		Additive	757/606	1.45 (1.06–1.99)*	68.3
Caucasian	All relevant articles	Dominant	1013/995	0.82 (0.65–1.02)	45.8
		Recessive	1013/995	1.10 (0.79–1.54)	57.2
		Additive	1013/995	0.95 (0.75–1.22)	68.2
	Excluded for DHWE	Dominant	817/761	0.71 (0.40–1.25)	71.3
		Recessive	817/761	0.97 (0.58–1.62)	73.0
		Additive	817/761	0.87 (0.59–1.30)	81.5
DR	All relevant articles	Dominant	952/749	1.13 (0.71–1.82)	69.2
		Recessive	952/749	1.49 (0.94–2.37)	73.8
		Additive	952/749	1.26 (0.89–1.79)	81.2
DN	All relevant articles	Dominant	818/852	0.91 (0.71–1.16)	0.0
		Recessive	818/852	1.25 (1.02–1.55)*	0.0
		Additive	818/852	1.07 (0.93–1.22)	0.0

Abbreviations and definitions: DHWE: deviated from HWE in cases and/or in controls; DR, diabetic retinopathy; DN, diabetic nephropathy.

* *P*<0.05.

After exclusion of the article deviating from HWE in cases and in controls, the A allele was still found to be significant associated with an increase risk of DMI in a recessive model (OR 1.38, 95% CI 1.00–1.91, *P* = 0.05). The dominant model and the additive model were remains non-significant (*P* values>0.05).

#### Subgroup analyses

In the subgroup analyses by ethnicity, significant associations between the A allele and increased risk of DMI were found among Asians both in a recessive (OR = 1.79, 95% CI 1.13–2.81, *P* = 0.01) and an additive (OR = 1.45, 95% CI 1.06–1.99, *P* = 0.02) model, but not in a dominant model (OR = 1.32, 95% CI 0.91–1.91, *P* = 0.14). However, the significant association between the *ICAM-1* K469E gene polymorphism and DMI was not found among Caucasians (Table 2).

With regard to the specific type of DMI, we also conducted the subgroup analyses stratified by DN or DR. The results showed that the A allele was found to be significantly associated with an increase risk of DN in a recessive model (OR = 1.25, 95% CI 1.02–1.55, *P* = 0.04). Moreover, the heterogeneity of the subgroup disappeared (*I* ^2^ = 0.0%). However, in the subgroup of DR, no significant association with the A allele was found in all genetic models (all *P* values>0.05).

#### Publication bias

**Figure 3:**
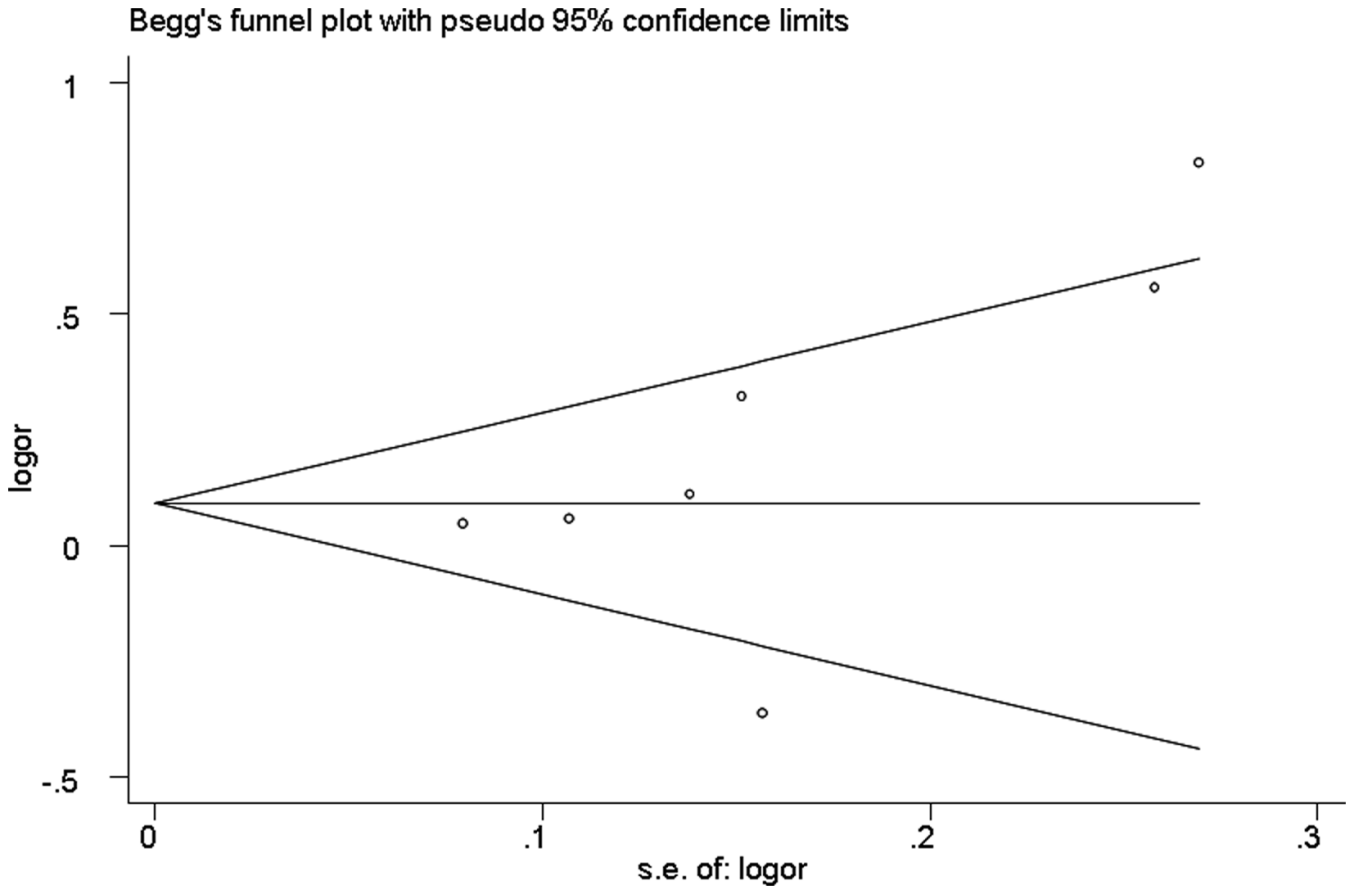
Funnel plot of publication bias for the *ICAM-1* gene K469E polymorphism.

The publication bias of the included studies was assessed by the Begg’s funnel plot and Egger’s test. The funnel plot for additive model in all studies showed no evidence for asymmetry (Figure 3). And Egger’s linear regression test revealed no significant publication bias was observed (*P* = 0.22).

## Discussion

The incidence of microvascular complications is increasing among the growing number of patients suffering from diabetes mellitus [32], [33]. The prevalence of overall microvascular complication is 30% to 40% at the time of diagnosis [34]. Although microvascular complications are the results of multiple causative factors, the genetic contribution of the individual plays a significant role in determining susceptibility to DMI [35]. The *ICAM-1* gene, encoding intercellular adhesion molecule 1, might play a crucial role in the pathogenesis of DMI including DN and DR [13], [36], [37]. The association between *ICAM-1* gene K469E (rs5498) polymorphism and DMI risk was first reported in an East Asian population [23]. However, as discussed above, conflicting data regarding the role of *ICAM-1* gene K469E polymorphism in DMI susceptibility have been reported [24], [25], [26], [27], [28], [29]. Against this backdrop, we performed a meta-analysis to clarify the relationship between *ICAM-1* gene K469E polymorphism and DMI risk.

This current meta-analysis of 7 studies including 1810 DMI cases and 1601 diabetic controls systematically evaluated the association between *ICAM-1* gene K469E polymorphism and DMI risk. With the use of results provided in previous studies, we compared the minor allele to major allele in additive model, recessive model and dominant model. A significant relationship was found between *ICAM-1* gene K469E polymorphism and increased DMI risk under a recessive model (OR = 1.37, 95% CI 1.04–1.80; *P* = 0.02). However, no significant association between *ICAM-1* gene K469E polymorphism and DMI risk was found in dominant and additive models (OR = 1.02, 95% CI 0.77–1.36; *P* = 0.88; OR = 1.17, 95% CI 0.95–1.43; *P* = 0.13, respectively). Furthermore, ethnic-specific meta-analysis showed that no significant association existed between *ICAM-1* gene K469E polymorphism and DMI in Europeans (all *P* values>0.05). In contrast, DMI risk was increased in Asians in recessive and additive models (OR = 1.78, 95% CI 1.13–2.81, *P* = 0.01; OR = 1.45, 95% CI 1.06–1.99, *P* = 0.03, respectively), suggesting a possible influence among environmental exposures and different genetic backgrounds. In the stratified analysis by the specific type of microvascular complication, the significant association was observed in DN subgroup in recessive model (OR = 1.25, 95% CI 1.02–1.55; *P* = 0.04). Nevertheless, no significant association of *ICAM-1* gene K469E polymorphism with DR subgroup was found (all *P* values>0.05). Since this meta-analysis included only two studies using DN population, the false positive association between *ICAM-1* gene K469E polymorphism and DN could not be ruled out because studies with small sample size may have random error to detect a slight effect. Additional future studies should be performed to focus on DN population. More studies are warranted to further validate microvascular complication type difference in the effect of this polymorphism on DMI susceptibility.

The polymorphism K469E (rs5498) is a non-synonymous single nucleotide polymorphism and resides in the fifth immunoglobulin-like domain of *ICAM-1* that is essential for dimerization, surface presentation and solubilisation of the protein [38]. It results in a change in the amino acid sequence of the Ig-like domain 5 (from glutamic acid to lysine). This domain is of crucial importance for the activity of ICAM-1 protein in its interactions with LFA-1 and for the adhesion of B cells [39]. Previously study has demonstrated that this polymorphism affects *ICAM-1* mRNA splicing pattern and TPA-induced apoptosis [40]. A recent genome wide association study has demonstrated a strong correlation between K469E polymorphism and sICAM-1 levels [20]. Thus, the K469E polymorphism of *ICAM-1* gene may play an important role in inﬂammation and atherosclerosis.

As the publication of findings often depends on the expectation of researchers, false-negative results may be suppressed or false positive results magnified [41]. Although the results of this study did not show significant publication bias, the number of studies included in this meta-analysis was small and large inter-study heterogeneity was observed. Significant heterogeneity existed in overall comparisons in each genetic model. The observed heterogeneity could be attributed to differences in several factors such as microvascular complication type, diabetes duration, ethnic variations, environmental factors and methodological factors in design and conduct of the studies. In addition, DMI is a complex etiology generated by combined effect of genetic and environmental risk factors. Due to different racial or ethnic populations with different frequencies of alleles, different genetic backgrounds may affect DMI susceptibilities. It has been reported that the K allele was risk factor for DR in Asian, whereas the E allele was risk factor for DR in Caucasian. This may reflect the genetic heterogeneity between different ethnicities.

The present meta-analysis had several limitations that must be taken into account. Firstly, only published data which were included by the published studies and unpublished studies which had null results were missed, which might bias the results, while our statistical tests may not have totally shown it. Secondly, the overall outcomes were based on individual unadjusted genotype data, while a more precise evaluation should be made by adjusting other potentially suspected factors including age, sex, and environmental factors. Thirdly, data were not stratified by other factors such as gender status and the clear diagnosis methods of DMI, because sufficient information could not be extracted from the original studies. Fourthly, because of the complex nature of DMI, it is unlikely that a SNP in one single gene would be obviously associated with an increase in DMI risk, without consideration of any other polymorphic susceptible genes. Fifthly, although there are many similarities in the coexistence of DR and DN being both as microvascular disease and microscopically both have capillary basement membrane thickening, they still have somewhat different pathogenesis. DR is characterized by a spectrum of lesions within the retina. These include changes in vascular permeability, capillary microaneurysms, capillary degeneration, and excessive formation of new blood vessels. However, the development and progression of DN is highly complex given the diversity of cell populations present within the kidney and the various physiological roles of this organ. High glucose concentrations induce specific cellular effects, which affect various resident kidney cells including endothelial cells, smooth muscle cells, mesangial cells, podocytes, cells of the tubular and collecting duct system, and inﬂammatory cells and myofibroblasts. Sixthly, both types of diabetes were enrolled in our study, and differences in pathogenesis between type 1 and type 2 diabetes are existed. Finally, since the number of studies included in the subgroup analyses was small, the results lacked sufficient reliability to confirm or refute an association in a definitive manner.

To our knowledge, this study was the first comprehensive meta-analysis to assess the relationship between *ICAM-1* gene K469E polymorphism and DMI susceptibility. It provided evidences of the association between *ICAM-1* gene K469E polymorphism and DMI risk, and supported the hypothesis that the *ICAM-1* gene K469E polymorphism might be a susceptibility marker for DMI. Since potential biases and confounders could not be ruled out completely in this meta-analysis, additional large case-control studies are required to validate our findings. Meanwhile, further studies regarding other SNPs (or haplotypes) in the *ICAM-1* gene and DMI susceptibility are also encouraged to help better understand the role of *ICAM-1* gene in DMI. Moreover, gene-gene and gene-environment interactions should also be considered in future studies.
